# The Diagnosis of Diseases of the Brain

**Published:** 1886-03

**Authors:** 


### Reviews and Notices of Books.

---

*The Diagnosis of Diseases of the Brain.* By W. R. Gowers, M.D. Pp. 246. 1885. London: J. & A. Churchill.

Dr. Gowers' former works are so suggestive and so useful, that we may fairly congratulate the profession that he has turned his attention to the diagnosis of diseases of the brain.

His book begins by a *resumé* of all that is known as to the motor path from the cortex, tracing the downward course from cortical centre by way of internal capsule, crus, pons, pyramids, lateral columns of opposite side. The ultimate destination of the sensory path is still to a large extent uncertain. Some fibres enter the optic thalamus, but their function is unknown, as there is not at present any evidence to show that impressions that influence consciousness pass through the thalamus, except perhaps in the case of visual impressions.

Accepting the view of the semi-decussation of the optic nerve, Dr. Gowers believes that it is in a high degree probable that (as Ferrier has suggested) in or near the angular gyrus there exists a visual centre, higher than the half-vision centre, in which the whole of the opposite field is represented. In this relation there must be a connection between this centre on one side and both occipital lobes, that with the opposite occipital lobe being probably by means of the fibres of the corpus callosum.

We know nothing of the cortical centre for taste, and even the nerve of taste is still somewhat uncertain. Dr. Gowers thinks that taste-impressions reach the brain solely by the roots of the fifth nerve, and that the roots of the glossopharyngeal have nothing to do with it.

In his remarks on the other cranial nerves he emphasises the fact that the facial nerve has no real origin from the nucleus of the sixth, although some of its fibres pass through it; that, alone of all the cranial nerves, the fourth nerves decussate between the surface attachment and the nucleus; and that there seems to be an extensive connection by large nerve-fibres, the posterior horizontal fibres, between the three nuclei for the nerves of the external ocular muscles, the third, the fourth, and the sixth.

The mode of connection of the cerebellum with the frontal, temporal, and occipital lobes of the brain,—regions in no way concerned with motor or sensory functions,—gives force to the old idea that the cerebellum is in some way concerned in intellectual processes.

Of the basal ganglia, the optic thalamus is concerned with some of the higher reflex processes. When the posterior portion of the thalamus, the pulvinar, is diseased, hemiopia occurs, possibly through interference with the posterior portion of the optic tract, which enters the corpus geniculatum close to the posterior extremity of the thalamus.

Of the corpus striatum, both portions, the caudate nucleus and the lenticular nucleus, have extensive connections with the cerebellum. In congenital absence, too, of the cerebellum, the corpus striatum is reduced to a third of its ordinary size. Dr. Gowers holds that no symptoms result from a lesion of either portion of the corpus striatum, so long as it does not interfere with the functions of the motor or sensory parts of the internal capsule. In this we are disposed to some extent to join issue with him. The fibres to the face, the arm, the leg, from the motor area of the cortex, are very closely associated in the lenticular nucleus; and pathological anatomy seems to show that plugging of any of the small terminal arteries in this situation leads to inco-ordination of movement. That it is not a motor centre is indeed probable: that its grey matter, alone in the cerebrum, should be without function, seems in the highest degree improbable.

Little is known of the function of the cerebellar hemispheres. Lesion of the middle lobe of the cerebellum, on the contrary, is associated with a peculiar unsteadiness of movement. This lobe is concerned with the maintenance of equilibrium, perhaps by combining the afferent impressions, and arranging for harmonious centrifugal impulses.

Dr. Gowers gives a clear account of the circulation in the brain, and remarks that whilst the cerebellar arteries communicate freely, and hence necrotic softening of the cerebellum is rare, the opposite is the case in the medulla and the pons. Our author goes on to analyse symptoms, and makes some preliminary remarks on mechanism. Symptoms may depend upon loss of function due to damage that falls short of destruction, such as compression, defective supply of arterial blood; such, too, as irritation, which may not only cause over-activity of nerve elements, but may lessen their activity and even arrest it. It is probable that the nervous system is full of mechanisms, whereby the action of certain centres is controlled by that of other centres; and it is probable that the chief mechanism of this association is control, and that what we call the excitation of one centre by another may be very often simply a lowering of control, permitting activity. Thus we can understand that inhibition, as well as excitation, may be a result of the pathological process that we call irritation.

Some symptoms, such as local palsy, are due to and indicate interference with the function of a definite part of the brain. These are termed "focal" symptoms, because they are due to disease at a given spot. Other symptoms, such as loss of consciousness, or delirium, indicate a wide-spread interference with the function of the brain, and are called "diffuse."

In discussing convulsion, the characteristic of the convulsions of organic brain-disease is their local commencement; and a local aura without convulsions has the same significance as a local spasm.

The most frequent seat of disease causing hemianæsthesia is the posterior part of the internal capsule. The additional presence of crossed amblyopia with hemianæsthesia will often show that the affection is hysterical, as crossed amblyopia dependent on organic disease would necessitate a very extensive brain-lesion, and would be evidenced by other symptoms. Dr. Gowers considers that in the only situation in which a lesion can cause hemianæsthesia, with affection of the special senses, the affection of sight is always hemiopia. One quotation may be given: "Affection of the sight of one eye may be due to disease at either extremity of the visual arrangement, the optic nerve, or the highest cortical centre."

The author's remarks on diplopia, too long for quotation, merit attention, as also those on the muscular mechanism within the eye. The four forms of this internal paralysis within the eye are rarely the result of focal lesion.

In facial palsy the palate is never paralysed, or hardly ever. The tests for deafness are interesting. Deafness from intracranial disease is usually due to disease of the nerve at the base of the brain, less frequently of its nucleus in the medulla. It very rarely results from disease of the auditory centre in the first temporo-sphenoidal convolution, and is then on the side opposite to the disease. Bilateral deafness may be due to damage to both auditory nerves, and Dr. Gowers has also known it to be produced by a tumour of the corpora quadrigemina damaging the upper layer of the tegmentum of each crus cerebri, in which the auditory path lies. From symmetrical disease of the cortical centres it is extremely rare. But deafness on both sides is frequently due to symmetrical labyrinthine disease. The observations on laryngeal palsy are eminently suggestive. After a brief reference to that peculiar nuclear palsy, bulbar paralysis, the author discusses some of the psychical phenomena. He, of course, ignores the term "serous apoplexy" as a condition that has no existence. The symptoms of apoplexy are those of lowered cerebral function. The upward action of the irritation of the lesion, inhibiting the highest centres, is probably the cause of the initial loss of consciousness. All lesions are effective in proportion to the rapidity with which they are produced.

Headache, aphasia, vertigo, vomiting, temperature, pulse, and disturbances of the respiratory, digestive, and urinary organs are all passed in review with reference to the localisation of a cerebral lesion; and a careful chapter follows on ophthalmoscopic changes, and this mass of data is brought to bear on the diagnosis of the seat of disease.

The later lectures are taken up with the diagnosis of the nature of the lesion. The frequency of rupture of a cerebral artery during sleep is remarkable. Perhaps it is determined by the loss, in the recumbent position, of the aid of gravitation to the return of blood from the head. Possibly, moreover, the contraction of the vessels, that is said to attend sleep, is of arteries smaller than those that are the seat of miliary aneurisms, and may even increase the blood-pressure in the latter, and help to determine rupture. Dr. Gowers lays stress on the frequency of thrombosis in the cerebral veins, as distinguished from the same condition in the sinuses.

The pathological diagnosis of acute and chronic lesions, especially of tumour, is contrasted. The differentiation of the various forms of paralysis, the question of cerebral localisation experimentally and clinically investigated, secondary degenerations, Flecksig's observations of the embryonic synthesis of the nervous centres, the knowledge of the results of the various diseases of the cerebral circulation, the great doctrine of inhibitory centres, the influence exercised by the vasomotors, are only some few points that we owe to the patient study of physiologists and physicians of the present generation. Amongst these workers Dr. Gowers holds no mean place. This useful book of his will well repay careful study. To many it will throw much light on a pathway that is often beset with difficulties. It must add to the reputation, already high, of its talented author; and no higher praise can be given to it.

